# Innovative chitin-glucan based material obtained from mycelium of wood decay fungal strains

**DOI:** 10.1016/j.heliyon.2024.e28709

**Published:** 2024-03-22

**Authors:** Dhanalakshmi Vadivel, Marco Cartabia, Giulia Scalet, Simone Buratti, Luca Di Landro, Alessandra Benedetti, Ferdinando Auricchio, Stefano Babbini, Elena Savino, Daniele Dondi

**Affiliations:** a Department of Chemistry, University of Pavia, Viale Taramelli 12, 27100, Pavia, Italy; b Department of Earth and Environmental Sciences (DSTA), University of Pavia, Via S. Epifanio 14, 27100, Pavia, Italy; c MOGU S.r.l., Via S. Francesco d’Assisi 62, 21020, Inarzo, VA, Italy; d Dep. of Civil Engineering and Architecture (DICAr), University of Pavia, Via Ferrata 3, 27100, Pavia, Italy; e Department of Aerospace Science and Technology (DAER), Politecnico di Milano, Via La Masa 34, 20156, Milano, Italy

**Keywords:** Myco-materials, Wood decay fungi, Flexible materials, Chitin-glucan-materials, Nanofiber papers

## Abstract

Fungi are an alternative source to animal-based chitin. In fungi, chitin fibrils are strongly interconnected and bound with glucans that justify the unique matrix. The present study aimed to extract chitin and glucans from the mycelium of several wood decay fungal strains in order to obtain flexible materials and to check correlations between chitin content and the mechanical properties of these materials. Five strains were chosen in consideration of their different cell wall chemical composition (high content of α-glucans, β-glucans or chitin) to evaluate how these differences could influence the mechanical and chemical characteristics of the material. The fungal strains were cultivated in liquid-submerged dynamic fermentation (both flasks and bioreactor). Chitin and glucans were crosslinked with acetic acid and plasticized with glycerol to obtain flexible sheets. *Abortiporus biennis*, *Fomitopsis iberica* and *Stereum hirsutum* strains were found to adapt to produce material with adequate flexibility. The obtained materials were characterized by Thermogravimetric analysis (TGA) for the understanding of the material composition. The material obtained from each species was mechanically tested in terms of tear strength, elongation at break, and Young's modulus.

## Highlights

• Preparation of elastomeric leather-like material derived from fungi.
• Improvement of the fungi cultivation technique by using bioreactors.
• A post-processing technique was developed to increase the elastomeric properties.
• Understanding the role of different biopolymers content to serve as elastomers.
• Reduction of the environmental impact of the leather industry by avoiding breeding and killing animals and toxic industrial processes like tanning.

## Graphical abstract

**Figure:**
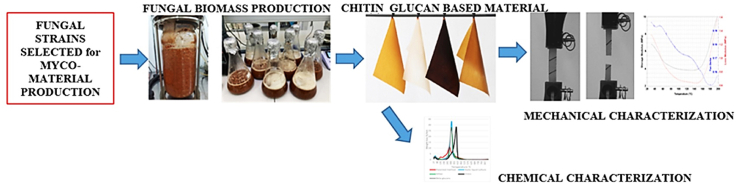

## 1 Introduction

Global environmental concern drives research to find bio-based alternative solutions to current polluting approaches. Both process and resulting product need to be the least energy-consuming and polluting as possible to be sustainable. In material science, the development of new biobased search for novel eco-friendly alternatives to fossil-based materials is recognized as a main way to improve sustainability [1] because of the ease of biodegradable products.

Chitin is the second most abundant natural polysaccharide globally available, second only to cellulose. Every year, 10^10^–10^11^ tons of chitin are naturally produced. Even if this polymer is useful for many applications, most of it is disposed of as waste [2,3]. Chitin is synthesized by arthropods and by fungi for structural support. Chitin is in crustacean chitin-based shells, insect exoskeletons and in the hyphal cell wall.

Chitin has been employed as functional bio-based material in food [4], agriculture [5], medicine [6], drug delivery [7] and water purification [8]. The principal chitin commercial sources are wastes from the shellfish industry like shrimp, crab and lobster shells where chitin content ranges between 8% and 40% [[9], [10], [11]].

Chitin sources like insects and fungi are getting more attention [12,13]. In recent years, fungi have been deeply investigated for industrial applications as bio-based materials [[14], [15], [16]]. The extraction of chitin from non-animal sources as fungi is possible [[17], [18], [19]] and it can be particularly attractive because it has no impact on animals [20]. Few cultivated edible species of fungi were already tested as chitin sources, with the aim of recycling waste parts. Wu et al. (2004) extracted a fungal chitin from *Agaricus bisporus* (J.E. Lange) Imbach, and its degree of acetylation (DA) ranged from 75,8 to 87,6%, which is similar to commercial chitin products [21]. Shiitake (*Lentinula edodes* (Berk.) Pegler) was also used to prepare fungal chitin [22].

In particular cases, fungal chitin produced better results when compared to traditional animal chitin. Chien et al. (2016) found that the chitin extracted from shiitake showed a better inhibitory effect on bacterial growth than the one extracted from crab shells [23]. Above all, in the most updated debates about plastic waste plastic production, thanks to the intrinsic bindings between fungal-chitin and glucans, this material has been recently taken into consideration as a novel bio-based nanofiber material [1].

Fungal-based materials cover a wide range of applications, as they can replace plastics, foams, timber, door cores, paneling, flooring, and other furnishings. Since these materials present low thermal conductivity, high acoustic absorption and fire safety properties outperforming traditional construction materials, such as synthetic foams and engineered woods, they are promising as thermal and acoustic insulation means [1].

The extraction of chitin from fungi has many advantages. Fungal chitin doesn't contain allergens like tropomyosin, myosin light chain and arginine kinase that are usually present in crustacean cuticles [24]. Moreover, the extraction conditions of chitin from mushrooms are milder than that from crustaceans because fungi contain lower mineral impurities associated with chitin and so the acidic extraction step to remove them is not required [1,20]. Also, the physic-chemical properties of fungal chitin could be well controlled because they can vary according to the fungal species, fungal strain, the harvesting period [21] and because mushrooms can be easily cultivated all year long with no seasonal fluctuations, without need of sunlight or any specific geographical restrictions. Finally, fungi generally include a substantial amount of β-glucans [25], which may be advantageous for the subsequent produced materials. It was in fact recently shown that chitin-based materials from fungal sources have superior tensile properties compared with their crustacean equivalents [26].

On the other hand, fungal chitin may have some disadvantages: fungal biomass has low chitin content and, as already said before, fungi do not synthesize pure chitin, but it is associated with glucans. This makes it more difficult to characterize, but this could represent a great and unique opportunity to select desired functionalities not only for material sciences speculations. The impurity of fungal chitin can be seen as an opportunity because glucan presence offers completely different structured and novel materials with new possibilities of application that can be explored [27]. Furthermore, the extraction process of fungal chitin needs to be scaled up to the industrial level and there is limited literature on this topic for myco-materials production [1].

In the past, it was proposed as the first application of this kind of material to use it as a skin substitute [28,29]. In recent years, further prospective applications of fungi-based materials, some of which have already reached the market, include paper (Nawawi et al., 2020), packaging and construction materials [30] or materials alternative to animal leathers [31,32]. Araki et al. (2012) prepared nanocomposite gels containing chitosan as a network polymer and chitin nano whiskers as reinforcing fillers, reaching Young's modulus of 169 kPa and stress at break of 135 kPa [33]. There are few studies in the literature concerning the mechanical characterization of chitin-based materials [30,[33], [34], [35]].

The present study aims to develop and characterize the mechanical performances of fungal chitin-glucan based materials obtained from fungal strains with different cell wall compositions, particularly given their application as viable alternatives to animal and synthetic leather in fashion or furniture products. The goal is also to evaluate, for each tested strain, the correspondence of the tested mycelium cell wall chemical composition to the final physic-mechanical properties of the obtained material. With this aim, an experimental campaign, involving tensile mechanical tests, thermal and environmental analyses was carried out to explore the possible attainable performances. The presented results are fundamental in identifying the properties of chitin-based myco-materials, to start with the design of targeted industrial products for selected applications. Based on obtainable properties, additional applications for these materials can be prospected and further investigated. Firm materials with rubber-like characteristics were obtained and selected, which could serve as viable alternatives to synthetic rubbers in packaging, flooring or sound/mechanical damping applications. It is interesting to observe that similar materials from fungi have raised the interest of Space Agencies given future space exploration and human establishments.

## 2 Material and methods

### 2.1 Fungal strain selection on cell wall polysaccharides content basis

Forested regions in northern Italy (the Lombardia and Piemonte Regions) have been surveyed to identify potential sources of samples for the isolation of Wood Decay Fungal (WDF) strains. As shown in Table 1, only five of the twenty-one strains were chosen for the current investigation based on the characteristics of each mycelium. Mycelia having higher growth rates and higher chitin content (as determined by TGA) were chosen for this study [36]. Based on the TGA data, Abortiporus biennis (064-18) and Fomitopsis pinicola (117-19) strains are distinguished by high α-glucan content, Fomitopsis iberica (104-19) by high β-glucan content and Coriolopsis gallica (086-19) and Stereum hirsutum (073-19) were chosen within a group with high chitin (weight loss percentage) content due to their higher growth rates [36].

**Table 1:** Fungal strains selected based on cell wall polysaccharides content [36].

Fungal species	Code	Cell wall polysaccharides content
*Abortiporus biennis* (Bull.) Singer	064–18	high in α-glucans
*Coriolopsis gallica* (Fr.) Ryvarden	086–19	high in chitin
*Fomitopsis iberica* Melo & Ryvarden	104–19	high in β-glucans
*Fomitopsis pinicola* (Sw.) P. Karst.	117–19	high in α-glucans
*Stereum hirsutum* (Willd.) Pers.	073–19	high in chitin

### 2.2 Fungal biomass cultivation

All the five tested strains were cultivated following the same procedure using a flask and bioreactor. For each strain, four plugs (0.5 cm^2^) were cut from actively grown cultures in 2 % malt extract agar (MEA - Biokar Diagnostic) and transferred into a flask containing 100 mL of sterilized liquid broth of malt (2 %), dextrose (0.5 %) and yeast extract (0.2 %).

After 7 days in a shaker incubator (181 rpm) at 25 °C in darkness, the biomass and the spent broth were blended in a sterilized metal blender and poured in a larger flask, containing 500 mL (for “flasks cultivation”) or 1000 mL (for “cultivation in bioreactor”) solution of the same broth. After three days, all the content of the flask was blended again in a sterile metal blender and, in case of “flasks cultivation”, 33 mL of the resulting cream were poured in each of 9 flasks containing 1.2 L of the same liquid broth and then they were placed in a shaker incubator (181 rpm) at 25 °C for 7 days. For the “cultivation in bioreactor”, all the resulting cream was poured in a 10 L bioreactor containing 10 L solution of the same broth. In this case, 2 mL of sunflower oil were added to limit the formation of foam. The bioreactor was set up at 25 °C and ran for 7 days with saturation of oxygen fixed at 50 %: this condition was maintained changing aeration 5–20 L/min and agitation of 80–150 rpm.

### 2.3 Alkali-insoluble molecules extraction and production of chitin-glucan based mats

The protocol used by Refs. [27,30] was followed with a slight modification. Once the biomass was obtained, it was washed with water to remove residues of the medium and extracellular compounds produced by the fungus and filtered through a nylon filtration bag. After washing, the fungal biomass was blended and kept in hot water at 90 °C for 1 h to remove water soluble compounds. After partial cooling down, the cooked biomass was filtered and washed again several times with nylon filtration bags to obtain a clean biomass. At the end of the last washing cycle, to recover it from the bag, the biomass was drained as much as possible and then resuspended in 1.5 L of water in a blender to be homogenized again. After that, 3.5 % w/w of pure NaOH was added and incubated for 2 h at 65 °C to eliminate proteins and alkali-soluble carbohydrates. After cooling to ambient temperature, the biomass was filtered again with nylon filtration bags. Several washing steps with water were necessary to restore pH to neutrality. The recovered drained biomass of alkali-insoluble molecules was resuspended in water. It forms the alkali-insoluble material (AIM slurry).

The last step consists in crosslinking the fibers. In 3 L of final volume 1 % of acetic acid and 10 % of glycerol were added to the AIM slurry. The mixture was blended to avoid the coagulation of the biomass and to ensure a homogeneous reaction. Then, the resulting cream was incubated in a water bath at 60 °C for 4 h.

After the crosslinking, the slurry was poured into a vacuum table (21 cm × 29.7 cm) prepared with a filter paper sheet. Once there was no more water dropping, vacuum was removed and the solid was transferred to a plastic foil. The filter paper was removed and the material was left to dry for at least 48 h until the piece was finally dry and self-supporting.

### 2.4 Thermogravimetric analysis (TGA) of the obtained material sheets

Thermogravimetric analyses were performed using a Mettler Toledo TGA1 XP1 thermogravimetric analyzer by heating the samples having the weight of 8–10 mg were inserted in an alumina crucible with a perforated lid at the temperature from 25 °C to 800 °C by the heating rate of 20 °C/min under N_2_ atmospheric condition with a mass flow rate of 100 mL/min. A blank performed with an empty crucible together with a perforated lid was subtracted from the curves [[37], [38], [39], [40], [41], [42], [43]]. Standards of β-glucan and chitin (both from Sigma Aldrich) were analyzed in the same conditions, allowing the comparison with the fungal mass loss at different temperatures. Thermogravimetric measurements were analyzed together with their first derivative (DTGA) to better estimate the temperature of the decomposition peaks (relative maximum of DTGA) and to locate the start/end of the decomposition (zeroing or flattening of DTGA).

### 2.5 Mechanical characterization of chitin-glucan based materials

**Fig. 1:**
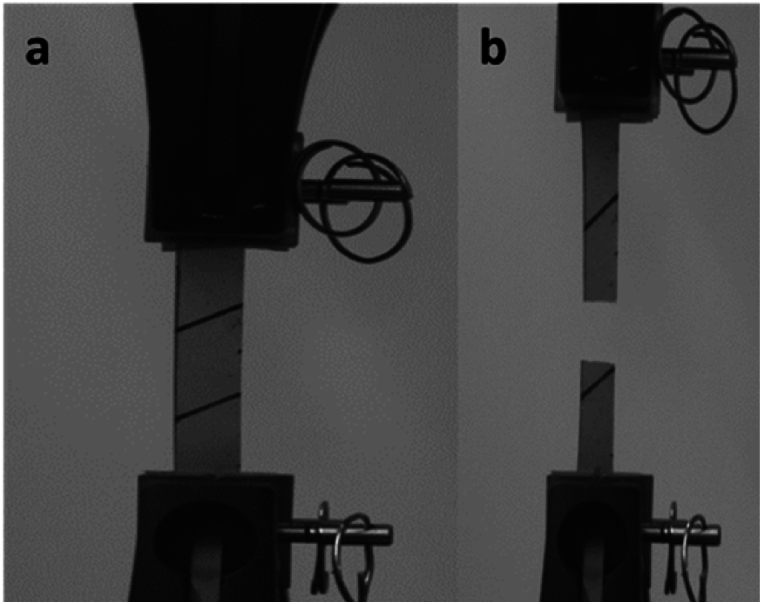
a) Clamped sample before and **b)** after the test. The images concern a specimen of the material obtained from *Stereum hirsutum* (Willd.) Pers. (073-19).

A MTS Insight Electromechanical Testing Systems 10 kN (MTS System Corporation) equipped with a 250 N load cell was used to conduct the tests. Tests were carried out at normal environmental conditions and under displacement control. The pneumatic grips were set 50 mm apart from each other. Dog-bone-like specimens indicated in the standard EN ISO 3376 are unsuitable for this type of materials. In fact, cracks can form at the curved parts of the sample during cutting. For this reason, rectangular specimens (110 mm × 10 mm) were used. A uniform speed of separation of the jaws of 2 mm/min was adopted, to ensure quasi-static conditions. For each material, six samples were tested until failure as shown in Fig. 1.

**Fig. 2:**
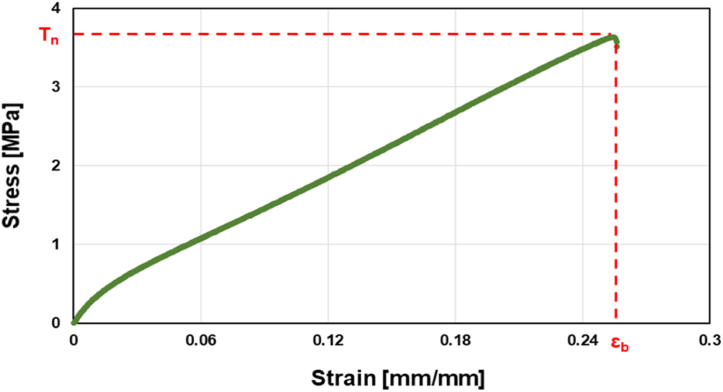
Representative stress/strain tensile test curve of the myco-materials.

Before testing, each sample was measured with a digital caliper and weighted to estimate its density. The data were recorded with TestWorks version 4 software and subsequently subjected to post-processing. Fig. 2 shows a representative uniaxial tension, stress/strain curve.

The tensile strength *Tn* (in MPa), the maximum stress a material can withstand during elongation before breaking is defined as

$$Tn=\frac{F}{wt}$$

where *F* is the highest force recorded during the test (in N), *w* is the specimen width (in mm) and *t* is the specimen thickness (in mm).

The percentage elongation εp to a specified load value is defined as

$$\varepsilon p=\frac{L-L_{0}}{L0}100$$

where *L* is the separation of the jaws and *L*_*0*_ is the initial separation of the jaws.

The elongation at break (*εb*) is the elongation in correspondence of *Tn*. Young's modulus *E* was calculated with the method of the secant modulus [44], considering the deformation range between 0 and 0.15 % of elongation with respect to the initial length of the specimen.

Elastomeric material obtained from *A. biennis* (064-18) fungal biomass in a bioreactor allowed to production of specimens with higher thickness and were tested by Dynamic Mechanical Analysis (DMA) (tensile and shear) tests. Specimens with 0.8 mm thickness were produced and employed in the tests. DMA allowed us to characterize the viscoelastic response of the material, in particular, the stiffness variation and the mechanical dissipation capacity as a function of temperature were measured.

DMA tests in tensile and in shear mode (plate-plate geometry) were performed by a TA 2980 Dynamic-mechanical analyser and by an AR2000ex Rheometer (TA Instruments) at 1 Hz frequency. Storage (E′, G’) and loss (E″, G”) moduli were measured as a function of temperature in the 20 °C/200 °C range; the mechanical damping factor tan δ was also measured.

### 2.6 Differential Scanning Calorimetry analysis (DSC) of the obtained material sheets

Differential Scanning Calorimetry (DSC) analysis was carried out on the same material by a DSC 2010 (TA Instruments) at the heating/cooling rate of 10 °C/min under a nitrogen atmosphere in the temperature range −50 °C/150 °C. A specimen of 12 mg was placed in Al crucibles and analyzed. Heating/cooling/heating/cooling cycles were applied to inspect the nature of the recorded thermal events; in particular, the presence and loss of absorbed moisture, as well as the moisture evolution process, were investigated by applying repeated heating/cooling cycles and by measuring the specimen mass before and after the test. Equal DSC tests applied to specimens from sheets of the same material did not evidence significant differences.

## 3 Results and discussions

Twenty-one fungal strains were characterized referring to cell wall composition through thermogravimetric analysis (TGA). These were selected out of all the fungal strains conserved in the Mogu company and isolated from wood decay fungi [36].

### 3.1 Fungal biomass cultivation

Biomass production in bioreactors is always higher than in flasks as shown in Table 2. In particular, the production is more than two times that of *A. biennis* 064-18 and *S. hirsutum* 073-19. The explanation may be attributed to the fact that cultivation in a bioreactor allows the monitoring of different parameters as pH, dissolved oxygen in the growing medium, aeration and agitation, thus creating the optimal conditions for growth. Data are reported as average ± 1 standard deviation, measured on 3 replicates.

**Table 2:** Fungal biomass production for the two different procedures.

Strain	Cultivation	Biomass production
		Dry biomass (g)
Abortiporus biennis (Bull.) Singer	Bioreactor	73 ± 1
	Flasks	38 ± 0.5
Fomitopsis pinicola (Sw.) P. Karst.	Bioreactor	37 ± 0.7
	Flasks	31 ± 0.6
Fomitopsis iberica Melo & Ryvarden	Bioreactor	51 ± 0.3
	Flasks	44 ± 0.5
Stereum hirsutum (Willd.) Pers.	Bioreactor	111 ± 1.2
	Flasks	60 ± 0.4
Coriolopsis gallica (Fr.) Ryvarden	Bioreactor	24 ± 0.8
	Flasks	25 ± 0.2

### 3.2 Alkali-insoluble molecules extraction and production of chitin-glucan based mats

The results obtained in the production of mats from the different sources and processes are reported in Table 3. During the treatment of the biomass obtained in liquid culture, problems occurred for *C. gallica* 086-19 and *F. pinicola* 117-19. The mycelium obtained from these two strains was composed of particles smaller than the pore size of the filter used, making the filtration and washing process impossible. It was observed that, regarding *S. hirsutum* 073-19, there was a higher solid reduction to have more AIM slurry material concerning the biomass obtained in bioreactor while for *A. biennis* 064-18 there was a higher solid reduction concerning the biomass obtained in flasks.

**Table 3:** Material production.

Strain	Cultivation	Percentage of solids reduction after heating & NaOH	thickness ± st. dev. (mm)	grammage (g/m^2^)
Stereum hirsutum (Willd.) Pers.	Bioreactor	82	0.3 ± 0.012	388.5
	Flasks	66	0.27 ± 0.01	328.5
Coriolopsis gallica (Fr.) Ryvarden	Bioreactor	nd	nd	nd
	Flasks	nd	nd	nd
Abortiporus biennis (Bull.) Singer	Bioreactor	59.4	0.793 ± 0.051	1048.2
	Flasks	68.25	0.265 ± 0.13	308.5
Fomitopsis pinicola (Sw.) P. Karst.	Bioreactor	nd	nd	nd
	Flasks	nd	nd	nd
Fomitopsis iberica Melo & Ryvarden	Bioreactor	43.02	0.298 ± 0.015	388.7
	Flasks	nd	nd	nd

*nd: not determined.

### 3.3 Mechanical tests

There are no standards currently available for the mechanical characterization of mycelium-based materials. So, a standard referring to animal leathers was selected as a reference for conducting the tests. EN ISO 3376 was adopted as a reference standard.

**Fig. 3:**
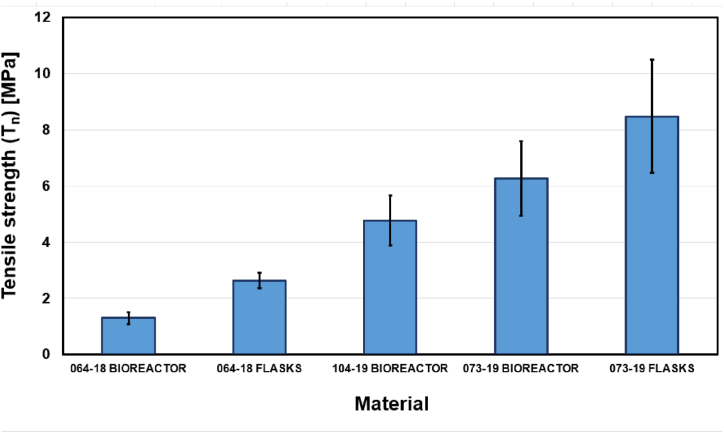
Maximal average tensile strength reported by chitin-glucan myco-materials.

**Fig. 4:**
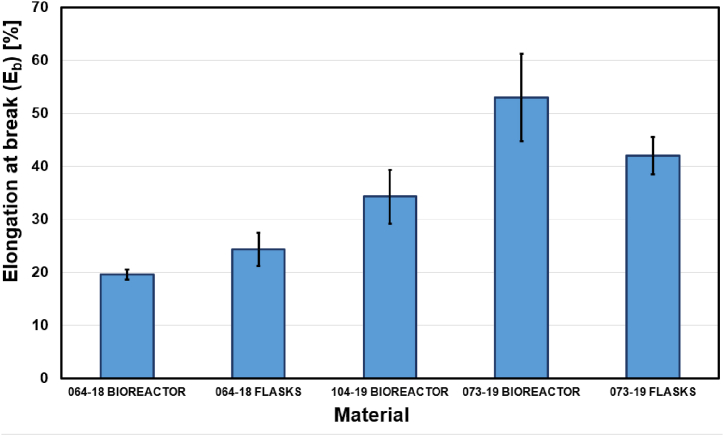
Maximal average elongation at break reported by chitin-glucan myco-materials.

**Fig. 5:**
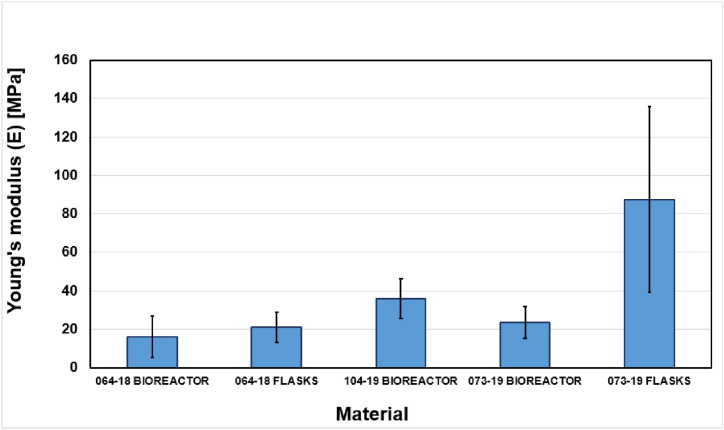
Maximal average Young's modulus reported by chitin-glucan myco-materials.

The values of tensile strength (*Tn)*, elongation at break (*εb)*, and Young's modulus (*E)*, obtained for each material group were reported in Fig. 3, Fig. 4, Fig. 5. The stress/strain curves were well reproducible for all the myco-materials.

The average tensile strength varied from 1.3 to 8.48 MPa which was shown in Fig. 3. The results show that the use of flasks instead of bioreactor increases the tensile strength by an amount of 1.3 MPa for the material obtained from *A. biennis* 064-18 and by an amount of 2.21 MPa for the material obtained from *S. hirsutum* 073-19. Among all, the highest tensile strength was achieved for *S. hirsutum* 073-19 grown in flasks, while *A. biennis* 064-18 cultivated both in bioreactor and flasks showed the lowest tensile strength.

The average elongation at break *εb* varies from 19.54 to 52.96 % which was shown in Fig. 4. Considering *A. biennis* 064-18, an increment of 5 % in the elongation at break is observed for the material obtained from mycelium cultivated in bioreactor compared to the material obtained from mycelium grown in flasks. Regarding *S. hirsutum* 073-19, a decrement of 11 % in the *εb* is observed for the material obtained from mycelium cultivated in a bioreactor compared to the material obtained from mycelium grown in flasks.

The material that stretches the most before breaking was obtained from *S. hirsutum* 073-19 biomass cultivated in a bioreactor.

The average Young's modulus varies from 16.21 to 87.47 MPa which was shown in Fig. 5. The material obtained from *S. hirsutum* 073-19 biomass cultivated in flasks was the stiffer and it showed high variability among the six tested samples.

The experimental setup was confirmed to be suitable and efficient for conducting tensile tests on these types of materials. Moreover, it was also confirmed that the fungal species as well as the cultivating procedure influence the mechanical properties. In particular, the materials produced with flasks appear more resistant to tensile tests. A comparison with the mechanical properties presented by similar materials as animal leather is quite difficult, considering the wide variability of thickness and properties presented by animal skins from different sources. However, in general terms, it can be observed that most of the tested materials present comparable strength, stiffness and ultimate elongation as animal skin, for cow/calf leather, reported values are in the range of 3–13 MPa for strength, 50–100 MPa for tensile modulus and 10–30 % for break elongation [45,46]. The data are reported as average ± 1 standard deviation, repetitions for each specimen is 5.

### 3.4 Dynamic mechanical analysis

**Fig. 6:**
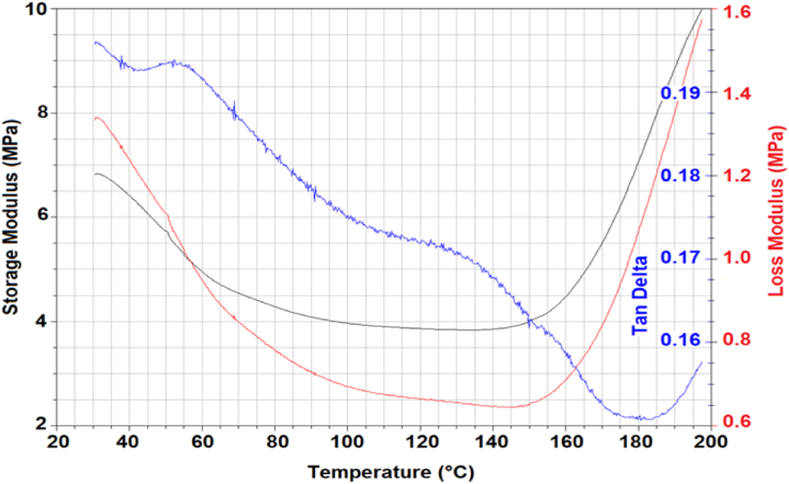
Elastomeric material from chitin extraction (*A. biennis* 064-18 cultivated in bioreactor). Dynamic mechanical properties (storage and loss moduli, tan δ) as function of temperature – tensile geometry.

**Fig. 7:**
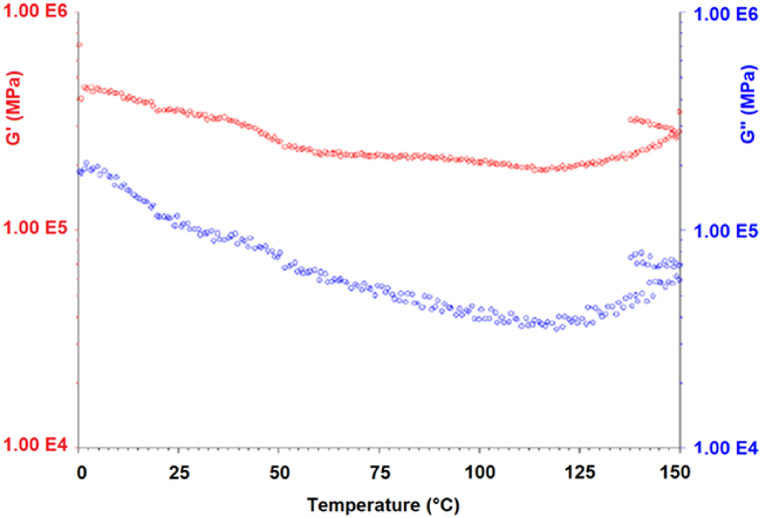
Elastomeric material from chitin extraction (*A. biennis* 064-18 cultivated in bioreactor). Dynamic mechanical properties (storage and loss shear moduli, tan δ) as function of temperature – plate-plate geometry. The upper points between 150 °C and 140 °C show the cooling results after heating.

DMA results of *A. biennis* 064-18 as a function of temperature showed that the tested material maintained a soft, elastomeric character from room temperature up to above 140–150 °C which was shown in Fig. 6 when an incipient stiffening effect occurs. This is possibly the consequence of absorbed humidity loss; as a matter of fact, a weight reduction of about 45 % was measured after desiccation at 150 °C. Loss of glycerol plasticizer may also occur in the high-temperature range. A similar behavior was observed also in a shear mode which, as expected, confirmed the persistence of higher stiffness also on cooling after the first heating ramp as shown in Fig. 7. It is interesting to observe that the damping factor tan δ is steadily in the range 0.15–0.20 which is consistent with the values usually presented by synthetic rubbers above glass-transition temperature.

### 3.5 Thermogravimetric analysis (TGA) of the obtained material sheets

**Fig. 8:**
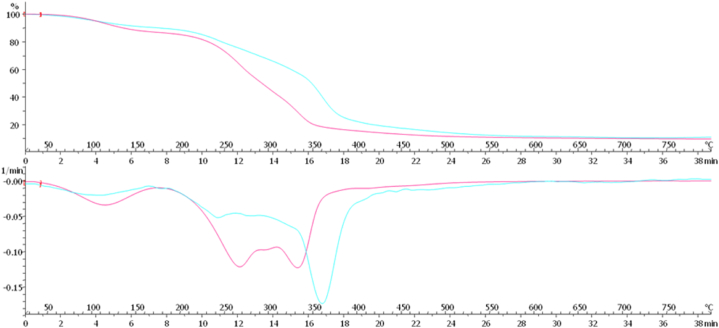
TGA curves showing the weight loss of the materials *A. biennis* 064-18 prepared using bioreactor (pink) and 073-19 prepared using bioreactor (cyan). (For interpretation of the references to colour in this figure legend, the reader is referred to the Web version of this article.)

**Fig. 9:**
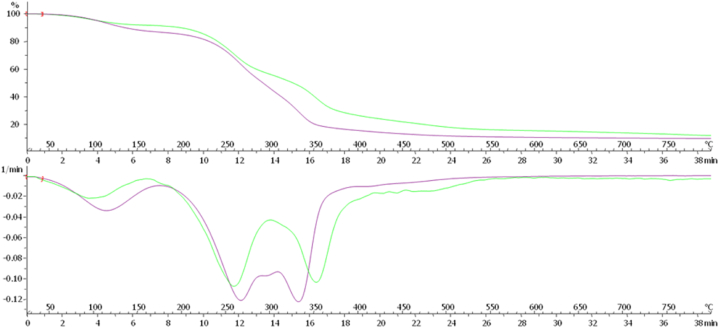
TGA curves showing the weight loss of the materials *A. biennis* 064-18 prepared using bioreactor (pink) and flask (green). (For interpretation of the references to colour in this figure legend, the reader is referred to the Web version of this article.)

Each material was analyzed by thermogravimetric analysis (TGA) to compare the initial composition of each mycelium (grown in liquid culture) and the corresponding resulting material after the processing. Strain diversity in the chemical composition of the materials was noted after the biomass processing. To explain the elastomeric properties of the materials key informative TGA results were shown in Fig. 8, Fig. 9. In general, α-glucan has the decomposition of at 250–350 °C and chitin has the decomposition of at 350–500 °C in TGA.

In Figs. 8, 064-18 using bioreactor (pink) showed less amount of chitin and a little more amount of α-glucan. But 073-19 using a bioreactor (cyan) showed a superior decomposition of chitin at 360 °C. For this reason, the material 064-18 prepared by using a bioreactor showed more flexibility due to the presence of α-glucan content with chitin.

In Figs. 9, 064-18 using a bioreactor (pink) showed less amount of chitin and a little more amount of α-glucan by decomposition at 330 °C and 270 °C, respectively. Contradictorily, 064-18 using flask (green) showed less amount of α-glucan and a little more amount of chitin by decomposition at 250 °C and 350 °C, respectively. So, the material 064-18 prepared by using a bioreactor showed more flexibility when compared to the material prepared using a flask. It has coherence with the dynamic mechanical analysis (DMA).

TGA revealed the presence of α-glucan content added with chitin polymer is highly necessary for the elastomeric properties of the materials derived from myco-materials that mentioned above. As a test, *A. biennis* 064-18 prepared by using a bioreactor as a material showing its superiority with its elastomeric properties due to the mixture of α-glucan with chitin.

### 3.6 Differential scanning calorimetric analysis (DSC) of the obtained material sheets

**Fig. 10:**
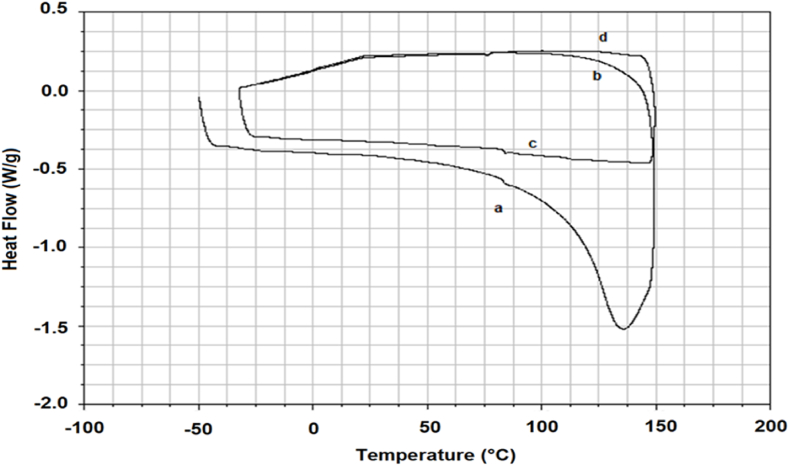
DSC tests of elastomeric material from chitin extraction (*A. biennis* 064-18 cultivated in bioreactor). a) and c) 1st and 2nd heating runs, b) and d) 1st and 2nd cooling runs (after heating).

With the knowledge of DMA and TGA results, we performed the DSC test of the material *A. biennis* 064-18 cultivated in the bioreactor. Fig. 10 showed an endothermic peak at 140 °C in the first heating run, which is no more present in subsequent cycles as shown in b, c and d.

This is an indication of absorbed water loss, supporting the previous observations of DMA results. However, the relatively high temperature of this loss, well above 100 °C, suggests that water molecules are possibly bonded to polar sites of chitin-glucan molecular structure and that the material can preserve a stable elastomeric character over a wide temperature range.

From an industrial point of view, it has been observed that the benefits of the fungus-derived material over the other leather substitutes include the simplicity of biomass cultivation (fungi can be grown under controlled fermentation conditions), the fungi's shorter growth period, and the lower carbon footprint [47]. In this research work, most strains that we discussed here are well-known for enzyme production and were less known for their application in bio-based materials. Species such as *Fomitopsis iberica*, are not known for either enzyme production or use in bio-based materials. In the recent study by Elsacker et al., the bulk of the 69 fungi included were engaged in various patents about fungal leather replacements. Materials made from these fungal groups are thought to be easily biodegradable and compostable after their useful lives, promoting a circular economy and reducing waste production [48].

## 4 Conclusions

In general, fungal strain selection depends on the high content of target biopolymers. But, in real-time applications, for leather-like materials in large-scale production, fungal strain growth rates, biomass production in liquid shaking culture and biomass loss during the extraction treatment are also important. The production method of mycelium in flask and bioreactor shows the difference in polymer content. *Abortiporus biennis, Coriolopsis gallica, Fomitopsis iberica, Fomitopsis pinicola and Stereum hirsutum* fungal strains were selected based on cell wall polysaccharides content. The strains are analyzed by DMA, TGA and DSC to understand their properties to act as elastomers.

Tensile tests showed that the different fungal species affect the material mechanical characteristics within the same strain. The mechanical testing campaign showed that the materials obtained from *S. hirsutum* 073-19 mycelial biomass are the most resistant (till 8.48 MPa before breaking) but also the most elastic (42–53 % stretching) while the materials obtained from *A. biennis* 064-18 mycelial biomass with the highest α-glucan content and the lowest chitin content, are the less resistant (1.3–2.6 MPa). In the production of leather-like materials, materials that could have elastomeric properties are in demand due to their flexibility in real-time applications. The thermogravimetric analysis (TGA) and differential scanning calorimetric analysis (DSC) strongly support the elastomeric properties of the material depending on the presence of α-glucan with chitin. Further, DSC explains the presence of water molecules that could be linked in between α-glucan and chitin. In addition, mycelium grown in a bioreactor produces a more elastomeric material than the one cultivated flask, where a little more chitin is present. This may be attributed to the fact that cultivation in a bioreactor allows the monitoring of different parameters as pH, dissolved oxygen in the growing medium, aeration and agitation, thus creating the optimal conditions for growth.

In literature, there are few works concerning the mechanical characterization together with thermal characterization of myco-materials. For this reason, the present work is very relevant for the elastomeric materials (leather-like) innovation and progression. The results obtained enrich the knowledge and consequently the potential input for this class of materials. Thus, we achieve renewable green materials for blue Earth.

Both animal leathers and their synthetic substitutes present heavy environmental issues, which, in the case of animal leather, are mostly related to the use of critical chemicals and the production of highly polluting process wastes. Synthetic alternatives, usually based on PVC and polyurethane, present some economical and durability advantages, although usually less performing from the mechanical, technical and sensorial point of view; however, they are mostly produced from non-renewable and non-biodegradable sources and, again, can be environmental and public health implications related to the manufacturing procedures and to the toxicity of the released and disposed products. In this context, fungi-based materials represent a valid natural alternative; they allow the control and the selection of required mechanical and physical characteristics over a wide extent by a proper definition of the production procedure. Further extension of the range of attainable mechanical response in fungi-based products can be expected by reinforcing the material with natural fibers or fabrics. Further developments, particularly addressed to enhance mechanical strength and control of water absorption may substantially improve their performances as leather-like materials.

## Funding

This project has been funded by 10.13039/501100002803Fondazione Cariplo, grant n° 2018-1765 entitled “Myco-advanced leather materials (MATER).” This is part of the project NODES which has received funding from the MUR – M4C2 1.5 of PNRR funded by the European Union - NextGenerationEU (Grant agreement no. ECS00000036).

## Availability of data and materials

The datasets used and/or analyzed during this study are available from the corresponding author on reasonable request.

## Ethical approval

Ethical approval is not required.

## CRediT authorship contribution statement

**Dhanalakshmi Vadivel:** Writing – review & editing, Writing – original draft, Visualization, Investigation, Formal analysis. **Marco Cartabia:** Data curation. **Giulia Scalet:** Methodology. **Simone Buratti:** Investigation. **Luca Di Landro:** Supervision. **Alessandra Benedetti:** Data curation. **Ferdinando Auricchio:** Validation. **Stefano Babbini:** Resources. **Elena Savino:** Methodology. **Daniele Dondi:** Supervision, Funding acquisition.

## Declaration of competing interest

The authors declare that they have no known competing financial interests or personal relationships that could have appeared to influence the work reported in this paper.
